# Fight or Flight? Curvilinear Relations between Previous Cyberbullying Victimization Experiences and Continuous Use of Social Media: Social Media Rumination and Distress as Chain Mediators

**DOI:** 10.3390/bs12110421

**Published:** 2022-10-30

**Authors:** Chenyu Gu, Shiyu Liu, Subai Chen

**Affiliations:** School of Journalism and Communication, Xiamen University, Xiamen 361005, China

**Keywords:** social media cyberbullying, continuous use of social media, social media rumination, distress, curvilinear relationship

## Abstract

Recently, the number of active users of social media platforms is declining, posing a challenge to the sustainability of interest in social media and related industries. Therefore, it is of great significance to examine the environmental and psychological factors that influence the continuous use of social media. Until recently, little research has examined this topic from the perspective of the relationship between previous cyberbullying victimization experiences (PCVE) and the continuous use of social media (CUOSM), not to mention the psychological mechanisms that lead to this relationship. In addition, there are paradoxes in existing studies: one side believes that PCVE causes users to become addicted to using social media, while the other side argues that PCVE drives users to escape from using social media. In order to respond to this controversy and clarify the relationship between PCVE and CUOSM, this study introduces two psychological variables, namely “social media rumination (SMR)” and “distress”, in order to construct a chain mediation model. Researchers surveyed 692 people who had experienced social media cyberbullying, and analyzed the data through SPSS and Mplus. The findings were as follows: 1. There is an inverted U-shaped curve relationship between PCVE and CUOSM. Specifically, the relationship initially exhibits a positive correlation (the period named fight), which then becomes negative (the period named flight). 2. When PCVE and CUOSM are positively correlated, SMR is the main factor that contributes to an increase in CUOSM. 3. When PCVE and CUOSM are negatively correlated, distress is the major factor that causes a decline in CUOSM. This study provides an explanation for the controversy in previous research, expands the scope of social media research, and provides a practical reference for social media platforms to enhance their existing users’ continuous use.

## 1. Introduction

Social media, an interactive platform that is based on mobile communication and Internet technologies, promotes the production, collaboration, and sharing of user content [1]. In addition, social media owns a large number of users and information content, which provide a suitable environment for the development of online games, corporate marketing, and mobile advertising, as well as a steady flow of traffic and profits for the stakeholders in the industry chain. However, in recent years, the development of social media has reached a bottleneck: many social media platforms have begun to experience a decline in active users. Meta recently released data for the second quarter of 2022, showing that the number of global monthly active users (MAU) of its Facebook platform was 2.93 billion, with 1.96 billion daily active users (DAU), a decrease of 200 thousand compared with the first quarter. The decrease in the number of active Facebook users caused Meta’s advertising revenue to decrease to some extent. For the first time in this quarter, revenue decreased year-on-year, and net income also decreased by 36%. When the number of users who stop continuously using social media reaches a certain scale, it may cause social media to fail to achieve sustainable development, and further affect the development of social media-related industries [2]. Therefore, it is of great significance to investigate the factors that influence users’ continuous use of social media.

Factors that have been reported to influence the continuous use of social media mainly include the following: privacy concerns [3], fatigue [4], overload [5], subjective norms [6], etc. However, there is a gap that few studies have focused on, which is the relationship between previous cyberbullying victimization experiences and continuous use of social media. Moreover, the existing research on this topic reached controversial findings: on one hand, some studies argued that previous cyberbullying victimization experiences exacerbate social media users’ online addiction, which means they will spend more time using social media continuously [7]. On the other hand, some studies found that previous cyberbullying victimization experiences make people use social media less continuously [4]. Therefore, the study raises two research questions: (1) How do previous cyberbullying victimization experiences influence users’ continuous social media use? (2) What are the mechanisms of this influence? In order to find an explanation for the controversy that previous researchers raised, the study carried out a survey among a special sample of 692 social media users who experienced cyberbullying victimization.

The current study revealed an inverted U-shaped curvilinear relationship between previous cyberbullying victimization experiences and the continuous use of social media. In the early stage, when the number of previous cyberbullying victimization experiences that a user has is at a low degree, individuals choose to “fight” as a result of social media rumination, leading them to become addicted to rethinking and managing their social media posts, thus increasing their continuous use of social media. However, the more social media cyberbullying they experience, more distress they suffer, driving people to retreat from using social media, which is the option of “flight”. In addition, the study constructed a chain mediation model (social media rumination and distress as mediators), which was applicable to the “flight” phase, and partially accepted in the “fight” phase, as the mediation effect of SMR was significant, while that of distress was not.

The contribution of the current study is to innovatively propose a nonlinear relationship between previous cyberbullying victimization experiences and the continuous use of social media, which provides a valid explanation for the controversy among previous studies. Moreover, this study once again verified that when receiving the same stimulation, the behavioral decisions people make do not necessarily develop in the same direction, layer by layer; in other words, there may be a nonlinear relationship. By introducing SMR and distress as mediators, this study explained the nonlinear relationship between the previous cyberbullying victimization experiences and the continuous use of social media. The current study not only broadens the scope of research on sustained social media use, but also offers a practical reference to sustain social media development.

## 2. Theoretical Framework

### 2.1. Continuous Use of Social Media (CUOSM)

The adoption and use of social media can be divided into two phases: first, initial adoption and use; second, continuous use. While initial adoption is important for the expansion of social media platforms, research in the field of information systems suggests that continuous use is the key to a technology’s success [8]. Although the initial use of a social media platform can recover the costs of investment to some degree, it should be noticed that the cost of attracting a new user is five times higher than that of retaining an existing user [9]. Consequently, for social media platforms, their profitability depends more on users’ continued use. However, after the explosive growth of social media, its “dark side” has been revealed in recent years [10]. Studies have shown that users are becoming bored of, and resistant to, mobile social media [11], leading to a loss in sustained users. This has become a crisis that social media platforms need to face [12]. Losing sustained users means losing sources of profitability, thus leading to failures in achieving expected revenues. Therefore, research on continuous usage is necessary for the development of social media.

The main theories that have been applied to analyze the continuous use of social media include: the expectation–confirmation model [13], uses and gratifications theory [14], theory of planned behavior [15], customer value theory [16], technology acceptance model [17], and information system success model [18]. In reviewing the literature, a trend can be found, in which factors such as emotions and personal psychological traits are attracting researchers’ attention. The current study, after summarizing the previous research, found that the factors affecting the continued use of social media mainly include the value factor [19], switching costs [20], social influence [21], privacy risks [22], and habits [23]. Protection motivation theory suggests that before adopting risk-reducing behaviors, individuals will first assess the risk, then develop protective motivation, and finally make behavioral changes, with personal experience playing an important role in the assessment process [24]. Therefore, it can be assumed that the more negative social media experiences an individual accumulates in his or her memory, the more inclined they will be to believe that they will be victimized while using social media; thus, they develop protective behaviors, such as abstaining from using social media.

It has also been pointed out that negative experiences with social media use (such as privacy violations) could discourage users from continuing to use social media [25]. However, different opinions exist regarding the impact of previous cyberbullying victimization experiences on the continuous use of social media. Some studies have suggested that previous cyberbullying victimization experiences leads to social media addiction [26]. Although addiction is an abnormal psychological state, it does mean that users have a higher tendency to use social media continuously. However, there are also some studies that conversely argue that previous cyberbullying victimization experiences may cause people to flee social media [27]; the reason for this is something that this study attempts to respond to. 

Moreover, studies have proven that previous cyberbullying victimization experiences significantly affects individuals’ social media rumination and distress [28,29]. Meanwhile, social media rumination has been shown to be associated with continued use of social media [30], while distress has been verified to cause users to reduce their use of social media [31]. According to these findings, this study speculates that social media rumination and distress would affect the relationship between previous cyberbullying victimization experiences and the continuous use of social media. Therefore, this study also attempts to explain how previous cyberbullying victimization experiences affects the continuous use of social media, by introducing social media rumination and distress as mediating variables.

### 2.2. Hypothesis of Nonlinear Relationship between Previous Cyberbullying Victimization Experiences (PCVE) and Continuous Use of Social Media (CUOSM)

Cyberbullying is a way of inflicting emotional pain on others through information technology [28]. Common cyberbullying behaviors include spreading rumors, revealing personal information or photos of other people without permission, sending threatening messages, and publicly making fun of others [32,33]. Cyberbullying frequently occurs on social media platforms; a large number of social media users have experienced online violence [34]. Thus, it is necessary to explore cyberbullying in depth, especially in social media contexts. The effect of previous cyberbullying victimization experiences is an important topic in cyberbullying research. It has been found that PCVE can cause many undesirable psychological traumas to individuals [35], such as distress, depression, and fear [36,37]. In addition to causing psychological damage, previous cyberbullying victimization experiences can also lead to problematic behaviors in individuals, such as Internet addiction and sleep disorders [38]. Through a review of the literature, this study found that there are still gaps that could be bridged concentrating on the effect of previous cyberbullying victimization experiences on the continuous use of social media, namely the following: (1) there is a debate, mentioned above, over whether previous cyberbullying victimization experiences increases the continuous use of social media or decreases it. One side argues that previous cyberbullying victimization experiences increase the continuous use of social media [39,40,41], while the other side argues that they decrease the continuous use of social media [42,43]. (2) Much of the research that investigated the impact of previous cyberbullying victimization experiences on the continuous use of social media was based on linear relationships; however, studies have shown that people’s online behavior does not often follow a constant layer logic, which is to say there may be a nonlinear relationship between previous cyberbullying victimization experiences and the continuous use of social media. 

Social cognitive theory (SCT) is frequently used to explain an individual’s behavior. The theory assumes that there is a dynamic interaction among individual psychological states, the environment, and behavior. An individual’s behavior is influenced by both environmental and psychological states. Moreover, individuals actively relate their past experiences to their psychological states, and then apply them to decode information and make behavioral decisions. Social cognitive theory has been widely used in studies of cyberbullying and social media usage, in which previous cyberbullying victimization experiences are generally treated as the environmental stimulus and assumed to change individual behavior [44]. For example, a study by Cao et al. demonstrated that previous cyberbullying victimization experiences significantly reduced users’ social media usage behavior [27]. An individual’s behavior is the action taken to adapt to the situation, and is decidedly dependent on the individual’s past experiences. When having not experienced much cyberbullying (low PCVE), people tend to use the experiences of others as a reference, which may lead to a third-person effect that causes people to overestimate their ability to cope with cyberbullying [45]. Therefore, individuals are more likely to adopt proactive protective behaviors, such as arguing with others online [46], and rethinking their posts on social media platforms [47], which leads to more social media use, and even addiction. However, when people find that when they still cannot cope with cyberbullying after making efforts to actively resist it, avoidance can become another possible protective behavior [48]. When people experience more cyberbullying victimization (high PCVE), they are more able to objectively judge their own ability to cope with it. At the same time, more experiences means that individuals accumulate more negative emotions that it brings [49], which are an important reason for reducing their continued use of social media [50]. When people find it difficult to cope with the negative emotions that are associated with online violence, they may tend to protect themselves by avoiding the use of social media. 

Based on the logic demonstrated above, this study speculated that there may also be a nonlinear relationship between PCVE and CUOSM. People may try to ruminate and fight when they experience an initial increase in cyberbullying. Studies have shown that PCVE increases users’ social media rumination (SMR) and fear of missing out (FOMO) [51]. Specifically, there will be a higher trend in CUOSM, as people fear missing online content that is relevant to them, in addition to repeated thinking about what they posted on social media. However, as the PCVE increases, users perceive more negative emotions, such as stress and anxiety [52,53]; these negative emotions may, in turn, reduce the CUOSM [54]. According to what has been discussed above, this study suggests that there is a first positive, and then negative effect of PCVE on CUOSM, and proposed the following hypothesis.

**H1.**  *There is an inverted U-shaped curvilinear correlation between PCVE and CUOSM.*

### 2.3. Social Media Rumination (SMR) and Distress as Mediators

In order to further explain the mechanism, we introduced two variables, which are social media rumination (SMR) and distress, as chain mediators.

The study first explored the association between PCVE and SMR. Lannin suggested that social media rumination refers to a tendency to repeatedly think about one’s social media posts, relevant online contextual factors, and the consequences of those posts; this is thought to increase the usage of social media [55,56]. Cyberbullying, as a stressful event, may arouse rumination in victims. Response style theory suggests that individuals will adopt a certain “style” of response to these injuries, meaning that they will continue to focus on the stressor that caused the injury; in other words, experiences of cyber violence may exacerbate individuals’ ruminative thinking [57]. Feinstein et al. found that the psychological trauma that was caused by cyberbullying to victims was sustained over time, as a result of victims’ regurgitated thinking about the source of the harm [58]. There is also a lot of prior research that showed that exposure to a cyberbullying environment is one of the main predictor variables of rumination [59]. Individuals who experience cyberbullying repeatedly recall those scenarios, as well as related online content, such as posts on social media platforms, and consequently develop ruminative thinking; this is a mediating variable between the experience of cyberbullying victimization and negative outcomes, such as depression, frustration, and Internet addiction (social media overuse) [60,61]. All of the above suggests that individuals who suffer from cyberbullying victimization tend to show higher levels of rumination, leading to an increase in the individuals’ continuous use of social media. Thus, this study proposed the following hypothesis.

**H2a.**  *SMR mediates the effect of PCVE on CUOSM; an increase in PCVE leads to an increase in SMR, which in turn leads to an increase in CUOSM.*

This study hypothesized that social media rumination always positively mediates the effects of PCVE on CUOSM. While simultaneously hypothesizing that as experiences of cyberbullying increase and negative emotions accumulate, people will cope with these negative emotions through avoidance. Some studies have pointed out that distress is one of the important negative emotions that is associated with cyberbullying [62]. In order to avoid the distress and other mental stress that are caused by cyberbullying, people reduce their use of social media [63]. We then need to discuss the relationship among PCVE, SMR, and distress. Numerous studies have shown that cyberbullying leads to an increase in individual distress. For instance, a study by Cénat on a sample of 8194 adolescents in Quebec found that cyberbullying significantly predicted distress levels, even more significantly than the effect of offline bullying [64]; a study by Bauman and Newman obtained similar results, and also verified that the experience of cyberbullying exacerbates individual distress [65]. Therefore, this study concluded that there is a positive correlation between PCVE and distress. 

What, then, is the connection between SMR and distress? There are several studies that demonstrated that individual rumination level is a significant predictor of distress degree. A two-year follow-up survey of a sample of 1620 Swedish subjects by Mazzer et al. found a significant and stable association between rumination and distress, with rumination always significantly predicting distress [66]; similar results were obtained in a study by Lannin et al., based on social media contexts [57]. Therefore, it is suggested that there is also a positive correlation between SMR and distress. In summary, both PCVE and SMR are believed to increase distress levels among social media users [67]. When it comes to the relationship between distress and the continuous use of social media, in terms of the negative outcomes of distress, some studies have pointed out that excessive distress can lead to avoidance of the source of distress; when individuals experience more distress than they can tolerate, avoidance behavior occurs [68]. Analogously, in this study context, as the experience of cyberbullying victimization increases, the individual’s distress also increases, and when the individual’s tolerance level is exceeded, the individual may choose to flee from using social media, in order to avoid experiencing cyberbullying again. Therefore, it is suggested that there may be a negative association between distress and continued social media use. Based on the logic above, the current study proposed hypotheses as follows:

**H2b.**  *SMR plays a mediating role in the effect of PCVE on distress; an increase in PCVE leads to an increase in SMR, which in turn leads to an increase in distress.*

**H3a.**  *Distress plays a masking mediator role in the effect of SMR on CUOSM.*

**H3b.**  *Distress plays a masking mediator role in the effect of PCVE on CUOSM.*

**H4.**  *SMR and distress play a chain-mediating role in the effect of PCVE on CUOSM.*

### 2.4. Current Study and Conceptual Model

**Figure 1:**
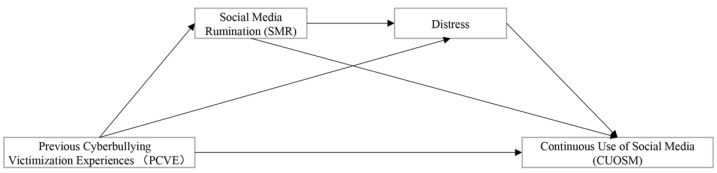
Conceptual model.

In order to better explain the mechanism of the effect of PCVE on CUOSM, this study proposed six hypotheses (as shown in Table 1), and built a chain mediation model (as shown in Figure 1). Whether this model applied to different stages of the effect of PCVE on CUOSM (positive and negative correlations) remained to be verified; therefore, this study also proposed an RQ: is the chain mediation model applicable at different stages of PCVE’s impact on CUOSM? Due to the nonlinear relationship between PCVE and CUOSM, this study performed a phased (left and right side of the curve inflection point) model test [69].

## 3. Materials and Methods

### 3.1. Participants and Procedures

“Questionnaire Star”, a professional survey distribution platform in China, was chosen to be the approach for this study to collect questionnaires. The selection criteria for the questionnaire were as follows: (1) For the purpose of this study, we only need to examine social media users who have experienced cyberbullying, so those who answered at the start claiming that they have not experienced cyberbullying will be excluded. (2) The IP address of the answering device was limited, in order to guarantee that every participant could not fill in the questionnaire twice. (3) Only those questionnaires that were successfully answered in an answer time that was longer than 60 s, were selected.

Participants completed the survey via computer and mobile devices. Following consent, participants answered questions that were related to the following scales. Two thousand questionnaires were sent out, and after excluding invalid questionnaires, which contained null data, we ended up with a sample of 692 participants who had experienced online violence. The qualification rate was 34.6%, and the basic demographic variables are tabulated in Table 2.

### 3.2. Measurements

The instrument of this study included measures of previous cyberbullying victimization experiences, social media rumination, distress, and continuous use of social media. The questionnaire for this study was designed on the basis of scales that were validated from previous studies; sub-items within each scale were averaged, resulting in composite scales. Before formal measurement, each variable was pre-tested, and the items with factor loadings below 0.5 and those with double factor loadings were removed, in order to obtain the formal questionnaire.

Previous Cyberbullying Victimization Experiences (PCVE): the previous cyberbullying victimization experiences questionnaire was used to measure the level of cyberbullying experienced by social media users [70]. The same five question items as the original scale were used in this study for measurement (for example, “Someone has posted comments that hurt me on the social media”). A Likert scale of 1 (strongly disagree) to 7 (strongly agree) was used to grade all of the responses; those with higher scores indicated more previous cyberbullying victimization experiences.

Social Media Rumination (SMR): the social media rumination questionnaire was used to assess the level of social media rumination [52]. The original version of the questionnaire had 12 items, but this study excluded the symptom regurgitation items, since they overlapped with depressive symptoms. Finally, five items were retained (for example, “do I worry about how people will react to my social media posts”). The responses were graded on a seven-point scale, ranging from 1 (not at all true of me) to 7 (extremely true of me), with higher scores indicating higher levels of social media rumination.

Distress: the distress scale was a three-item self-reported survey (e.g., “how often did you feel nervous, hopeless, so depressed that nothing cheered you up”) [71]. The responses were graded on a seven-point scale, ranging from 1 (never) to 7 (often), with higher scores indicating higher levels of distress.

Continuous Use of Social Media (CUOSM): the continuous use of social media questionnaire [51] was a self-reported questionnaire, and contained the same four measures as the original version (e.g., “I am willing to continue to use the current social media platform where I have suffered cyberbullying”). The participants were given a seven-point scale, ranging from 1 (strongly disagree) to 7 (strongly agree), with higher scores indicating higher willingness to continue using.

### 3.3. Data Analysis

Since the sample was sufficient, when a sample’s data contained missing values, we directly rejected that sample; hence, our final sample (*N* = 692) did not contain missing values. The validity and reliability of our questionnaire were tested using Mplus 8 and SPSS. Moreover, we referred to Robie and Ryan’s method to conduct a hierarchical polynomial regression analysis [72], in order to explore the nonlinear relationship between previous cyberbullying victimization experiences and the continuous use of social media. Moreover, the PROCESS macro for SPSS was used to evaluate the chain mediation model with bootstrapping (95 % CI, 5000 samples). Gender (1 = Female, 2 = Male), highest degree attained, age, and net age were among the covariates examined in this model.

## 4. Results

### 4.1. Measurement of Model

Table 3 shows that the McDonald’s ω and composite reliability of scales were higher than the acceptable value (>0.80). This means that the reliability was satisfactory. The factor loadings of all measure items on this study scale were higher than 0.6, indicating that all measure items met the retention criteria. The KMO values of each scale were also analyzed in this study, and the results showed that all scales had KMO values that were greater than 0.6, representing acceptable structural validity. In order to assess convergent validity, the AVEs of the variables were calculated. AVE is better when it is higher than 0.5, but 0.4 is acceptable, because Fornell and Larcker suggested that if AVE is less than 0.5, but composite reliability is higher than 0.6, the convergent validity of the construct is still adequate [73]. Lam et al. also explained and confirmed this in their research [74]. As Table 4 shows, discriminant validity was tested by comparing the square root of the AVE with the correlations of the researched variables. The square root of the AVE was greater than the correlations, indicating good discriminant validity.

The goodness-of-fit metrics were then evaluated. Confirmatory factor analysis (CFA) of our questionnaire produced satisfactory fit values for the one-dimensional factor structure, after including the error covariances in the model. (χ2/df = 2.393 < 0.3, RMSEA = 0.045 < 0.15, SRMR = 0.039 < 0.05, GFI = 0.964 > 0.9, CFI = 0.970 > 0.9, NFI = 0.950 > 0.9, and IFI = 0.970 > 0.9).

### 4.2. Statistics

Table 5 shows the descriptive statistics and correlation analysis results. The previous cyberbullying victimization experiences (PCVE) was negatively associated with the continuous use of social media (CUOSM), but was positively correlated with social media rumination (SMR) and distress. The social media rumination (SMR) was positively correlated with distress and continuous use of social media (CUOSM). In addition, there was a negative correlation between distress and continuous use of social media (CUOSM).

### 4.3. Relationship between PCVE and CUOSM

Since this study used hierarchical quadratic regression to test the curvilinear relationship, the PCVE was standardized to reduce the effect of multicollinearity before conducting the regression analysis. In order to answer H1, we performed a hierarchical quadratic regression test. The two regression models represented the meanings of the following:

Model 1. Examining the effects of PCVE and demographic variables on CUOSM.

Model 2. Comparing whether a linear or curvilinear relationship has more explanatory power for PCVE affecting CUOSM, after controlling for demographic variables.

In Table 6, the results are presented of a hierarchical regression on self-reported willingness to continue using their current social media platform. Gender, age, educational background, and net age were controlled for all models.

In comparing models 1 and 2, it was found that the PCVE^2^ has more explanatory power than the PCVE on CUOSM. Based on this, the current study further compared the fitted curves for the effect of PCVE on CUOSM, i.e., comparing linear and quadratic fitted models using curve estimation in SPSS regression analysis. The results showed that the explanatory power of the quadratic curve (R^2^ = 0.107) was better than that of the linear relationship (R^2^ = 0.019), because of the larger value of R^2^. Therefore, a curve model with the standardized PCVE was constructed with its quadratic term as the independent variable, and CUOSM as the dependent variable, based on the relevant data from model 2. From the coefficients of the quadratic term, the relationship between PCVE and CUOSM was a “U” curve, with a downward opening (β = 0.310, *p* = 0.000), which meant that as PCVE increased, CUOSM initially increased, and then decreased.

**Figure 2:**
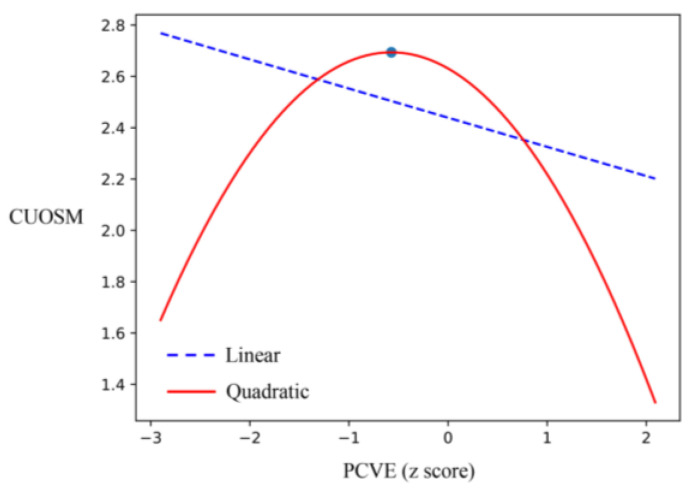
Relationship between previous cyberbullying victimization experiences (PCVE) and CUOSM (continuous use of social media).

As shown in Figure 2, the horizontal axis represents the standardized value of PCVE; there is an inflection point of trend change (−b/2a = −0.572). The left part of the curve (the z-value of PCVE goes from −2.90 to −0.572) revealed that there was a positive association between PCVE and CUOSM, indicating that social media users reported having more willingness to continue to use their current social media platform when they experienced more PCVE. The right part of the curve (the z-value of PCVE goes from −0.572 to 2.09) presents a negative association, revealing that social media users reported having less willingness to continue to use their current social media platform when they experienced more PCVE; thus, H1 held.

### 4.4. The Mediating Roles of SMR and Distress

Next, we further validated the applicability of the model at different stages of PCE impact on CUOSM. We divided the sample into two groups: the low PCVE group (the left part of the curve), and the high PCVE group (the right part of the curve), using the inflection point PCE = −0.572 as the cut-off point.

#### 4.4.1. Model Testing: The Left Part of the Curve

**Figure 3:**
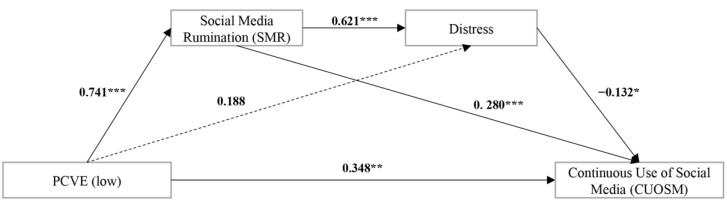
Analysis results: dashed lines represent a nonsignificant relation. *** *p* < 0.001, ** *p* < 0.01, * *p* < 0.05; PCVE = previous cyberbullying victimization experiences.

Table 7 shows the results of the polynomial regression analysis: PCVE (low) significantly and positively affected SMR levels (β = 0.741, SE = 0.137, *t* = 5.400, *p* = 0.000) and CUOSM (β = 0.348, SE = 0.123, *t* = 2.835, *p* = 0.005); meanwhile, there was not a significant relationship between PCVE (low) and distress (β = 0.188, SE = 0.162, *t* = 1.159, *p* = 0.248). Meanwhile, SMR positively predicted distress (β = 0.621, SE = 0.089, *t* = 7.001, *p* = 0.000) and CUOSM (β = 0.280, SE = 0.078, *t* = 3.636, *p* = 0.000) significantly. In addition, distress significantly and negatively affected the levels of CUOSM (β = −0.132, SE = 0.062, *t* = −2.132, *p* = 0.035). The specific impact of each path on the model is shown in Figure 3.

Next, we verified whether this chain mediating model held for the low PCVE group. The bootstrap test was performed using model 6 in the SPSS PROCESS macro, in order to assess the moderation effect. Before creating the interaction term, variables were concentrated. The results (as shown in Table 8) show that the following: 1, in the path of “PCVE → SMR → distress → CUOSM”, the chain-mediating effect of SMR and distress was not significant (95% boot CI = (−0.154, 0.005)); 2, in the path of “PCVE → SMR → CUOSM”, the mediating effect of SMR was significant (95% boot CI = (0.087, 0.368)); 3, in the path of “PCVE → distress → CUOSM”, the mediating effect of distress (95% boot CI = (−0.154, 0.005)) was not significant. In contrast, the total indirect effect was significant (95% boot CI = (0.005, 0.259)). In summary, we partially answered the RQ, that our model only partially held for “PCVE → SMR → CUOSM” in the group of social media users with low PCVE.

#### 4.4.2. Model Testing: The Right Part of the Curve

**Figure 4:**
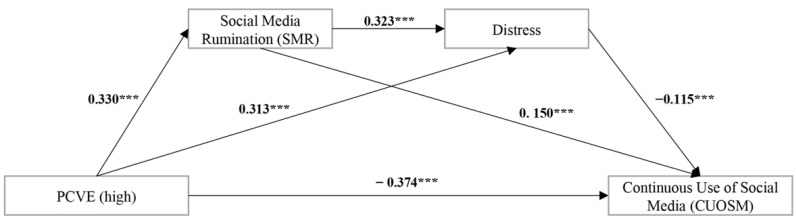
Analysis results: *** *p* < 0.001; PCVE = previous cyberbullying victimization experiences.

Table 9 shows the results of the polynomial regression analysis. PCVE (high) was significantly and positively affecting levels of SMR (β = 0.741, SE = 0.137, *t* = 5.400, *p* = 0.000) and CUOSM (β = 0.348, SE = 0.123, *t* = 2.835, *p* = 0.005), while there was not a significant relationship between PCVE (low) and distress (β = 0.188, SE = 0.162, *t* = 1.159, *p* = 0.248). Meanwhile, SMR positively predicted distress (β = 0.621, SE = 0.089, *t* = 7.001, *p* = 0.000) and CUOSM (β = 0.280, SE = 0.078, *t* = 3.636, *p* = 0.000) significantly. In addition, distress significantly and negatively affected levels of CUOSM (β = −0.132, SE = 0.062, *t* = −2.132, *p* = 0.035). The specific model is shown in Figure 4.

We then verified whether this chain mediating model held for the high PCVE group. The bootstrap test was performed using model 6 in the SPSS PROCESS macro, in order to assess the moderation effect. Before creating the interaction term, variables were concentrated again. The results in Table 10 show the following: 1) In the path of “PCVE → SMR → distress → CUOSM”, the chain-mediated effect of SMR and distress was significant (95% boot CI = (−0.024, −0.004)). 2) In the path of “PCVE → SMR → CUOSM”, the mediating effect of SMR was also significant (95% boot CI = (0.022, 0.084)). 3) In the path of “PCVE → distress → CUOSM”, the mediating effect of distress (95% boot CI = (−0.065, −0.013)) was significant too. However, the total indirect effect was not significant (95% boot CI = (−0.035, 0.038)). This may be due to distress masking the effect of SMR, which echoes the logic of the previous H1, and also answers the RQ. In summary, our model held in the group of social media users with high PCVE.

## 5. Discussion

The conclusions for the hypotheses and the RQ of the current study are shown in Table 11, and next, we discuss our specific findings.

### 5.1. Paradoxical Interpretation of PCVE and CUOSM: A New Inverted U-shaped Relationship

This study explored the effect of PCVE on CUOSM, and found that the relationship between the two is not simply linear, but inverted U-shaped curvilinear. In comparing Figure 3 and Figure 4, it can be determined that the effect of PCVE on CUOSM was different at each level of PCVE (low vs. high). Specifically, in the first (PCVE low) stage, the path coefficient β = 0.348 for the effect of PCVE on CUOSM showed a positive correlation between the two. Particularly, in this stage, users had not yet experienced too much social media cyberbullying, so they were unable to objectively judge their own ability to respond to cyberbullying, and may have used the experiences of others as a reference. There may also have been a third-person effect at this point [46]. During this period, users showed a greater willingness to continue using social media, such as for arguing with others online, or increasing their online time in order to monitor for new cyberbullying content [75]. As the PCVE increased, users tended to review their social media homepage and posts, which made them spend more time using social media, leading to a higher level of CUOSM. This finding is consistent with that from previous research [76].

However, in the second stage, the path coefficient of the effect of PCVE on CUOSM, β = −0.347, showed a negative correlation between the two. As PCVE increased, users felt a great deal of pain caused by PCVE; once it exceeded the individual’s tolerance, it drove them to retreat. This shift in user response behavior occurred when PCVE (z score) = −0.572. The effect of past negative experiences on perceived vulnerability can be explained by the availability heuristic, which postulates that the more instances of an experienced event are retrieved from memory, the higher the level of perceived occurrence frequency. Thus, an individual’s cumulative experience with negative events could influence the perception of future risk vulnerability [77]. According to protective motivation theory, severity and vulnerability are factors that influence individual avoidance behavior. Therefore, it is common sense that the more experiences of social media cyberbullying victimization an individual has in their memory, the more inclined they will be to think that they will be victimized by the cyberbullying; thus, they will be more likely to reduce social media use, in order to avoid cyberbullying. In other words, their willingness for CUOSM decreases; this is also consistent with the findings of previous research [19].

Our study explains the paradoxical findings of previous studies and fills in the gap of part of the research that concentrated on sustained social media use. In addition, the current study suggests that when examining social media user behavior, we should not only consider it as an outcome variable, but also as a cumulative process of change in which behavioral decisions are influenced and constrained by various factors. This calls for more attention to the mental damage that users suffer from social media cyberbullying.

### 5.2. PCVE and CUOSM Positive Correlation Phase: SMR Leads to Continuous Use

In the positive correlation phase between previous cyberbullying victimization experiences and the continuous use of social media, our model is partially adapted. Social media rumination is the main factor (mediator) that contributes to this unhealthy and continuous use of social media. As social media users’ experience of cyberbullying victimization increases, their level of rumination increases significantly; this in turn, leads to more time spent reflecting on and observing the content of cyberbullying, and to an increase in the continuous use of social media, which is also consistent with previous findings [28]. However, at this stage, neither of the two mediating paths of distress is significant. Thus, this explains the mechanism behind the positive correlation between previous cyberbullying victimization experiences and the continuous use of social media.

Another interesting finding was the change in the effect between PCVE and SMR. In comparing Figure 3 and Figure 4, it was found that as PCVE increased, the path coefficient of its effect on SMR decreased (β value decreased from 0.741 to 0.330). Although PCVE was positively correlated with SMR, the growth of SMR levels among social media users slowed down, which may be related to desensitization effects. The term desensitization is derived from the concept of systematic desensitization therapy in clinical psychology; this refers to the phenomenon that when an individual is repeatedly confronted with a stimulus that causes anxiety, rumination, or fear, these negative emotional responses gradually subside [78]. This desensitization phenomenon has been applied to the study of game violence, and it has been shown that the stimuli from violence are attenuated by counter conditioning [79]. Therefore, a similar mechanism may exist for the effect of PCVE on SMR. As a result of the positive correlation between SMR and CUOSM, the slowdown in SMR growth may also be a factor in the shift in the correlation between PCVE and CUOSM. We also suggest that future research could delve into the desensitization effect in social media cyberbullying.

The results of our study also showed that there is a tolerance level for distress. Distress is mainly derived from social media rumination, and is not directly related to cyberbullying victimization due to the lack of experience in judging cyberbullying. In the process of social media rumination and the continuous use of social media, distress acts as a masking effect, and motivates users to reduce their use of social media in order to avoid receiving distress. At this stage, distress is still within the tolerance range; hence, previous cyberbullying victimization experiences show a positive correlation with the continuous use of social media. Therefore, in order to ensure the retention of social media users, social media platforms should identify and curb cyberbullying content as early as possible at this stage, so that the situation does not continue to develop to the point where users begin to flee from using social media.

### 5.3. PCVE and CUOSM Negative Correlation Phase: Distress Drives Users to Escape from Social Media

In the negative correlation phase of previous cyberbullying victimization experiences and the continuous use of social media, our conceptual model is fully adapted. At this stage, distress is the main factor (mediator) that causes people to flee from using social media. This is also consistent with the expectation of protective motivation theory, which refers to the circumstance that when people’s distress accumulates beyond tolerance, people tend to flee from the source of the distress. At this stage, previous cyberbullying victimization experiences and distress become significantly correlated, and an increase in previous cyberbullying victimization experiences leads to an increase in social media rumination, which in turn generates more distress. Under this double pressure, distress exceeds users’ tolerance range, and makes them give up resistance (fight) and choose to retreat from (flight) using social media.

This also echoes a recent study on JOMO (joy of missing out); more people are escaping the pain of social media by abandoning its continued use [4]. This phenomenon should draw the attention of social media platforms. Cyberbullying that occurs on social media platforms not only hurts users, but also undermines the sustainable development of social media platforms, to a certain extent. Therefore, the interests of users and platforms are related; in other words, for social media platforms, protecting their users from cyberbullying is protecting their own interests.

### 5.4. Implications and Limitaions

The theoretical and practical implications of this paper are mainly reflected in the following three aspects: (1) The current study proposed a new model of sustained social media use (inverted U-shaped curvilinear). This theoretical model, to a certain degree, bridges a gap in the research and resolves the debate in this topic. Through the empirical data, this study provides a new and more integrative explanation of the relationship between previous cyberbullying victimization experiences and the continuous use of social media. The proposal of two different previous cyberbullying victimization experiences levels contributes to the integration between different theories, and further deepens research in the field of cyberbullying. This study also provides inspiration for later researchers, such as whether the fatigue induced by cyberbullying can also be characterized by desensitization (i.e., marginal effect). (2) The present study further validates the different mechanisms of the effect of previous cyberbullying victimization experiences on the continuous use of social media, in two phases via a model. The introduction of SMR and distress as mediators further guarantees the explanatory power and scientific validity of the theoretical model, expanding the field of research on social media rumination; in addition, it suggests more perspectives on the psychological and emotional research of social media users for subsequent research on the continuous use of social media. For example, some psychological variables, such as FOMO (fear of missing out) and anxiety, can be applied to advance future research. 3) This study also provides insight into the sustainability of social media platforms. As a cyberbullying environment causes more users to escape from using social media platforms in the long term, social media platforms should strive to reduce the number of cyberbullying incidents that occur, and create friendlier online environments.

Although research on the topic of cyberbullying and the continuous use of social media is popular, especially in communication fields, a large number of research questions still remain unanswered. Although the present study achieves some valuable findings, it also has some deficiencies. Firstly, the current study was based on a cross-sectional design; therefore, it had a deficiency in the explanatory power of causality, especially with the causal relationship between distress and the continuous use of social media. Some studies pointed out that distress should be used as a consequence of social media use [80], while others suggested that distress is an antecedent of reduced social media use [32]. Unfortunately, our study cannot explain the causal relationship between the two variables well; thus, we suggest that future studies provide a more in-depth explanation of the causal relationship between the two through controlled experiments. Secondly, the current study revealed the mechanism by which the effects of SMR are masked through distress; however, there may be other variables, such as anxiety and depression, that can also cause users to abandon continuous social media use, which are worth exploring in future studies. Thirdly, current research provided a generalized test of the continuous use of social media platforms; whether users escape from the Internet world or switch to another social media platform remains to be determined in depth. Finally, the current study considered social media as a general concept; however, there are differences in user behavior across different social media platforms [81], which were not discussed in depth in this study. Therefore, we suggest that future research consider different types of social media as moderating variables for a more detailed study.

## Figures and Tables

**Table 1:** Hypotheses and the research question.

Hypotheses	Contents
H1	There is an inverted U-shaped curve relationship between PCVE and CUOSM.
H2a	SMR mediates (+) the relationship between PCVE and CUOSM.
H2b	SMR mediates (+) the relationship between PCVE and distress.
H3a	Distress mediates (−) the relationship between PCVE and CUOSM.
H3b	Distress mediates (−) the relationship between SMR and CUOSM.
H4	SMR and distress play chain-mediating roles in the influence of PCVE on CUOSM.
RQ	Whether the chain mediation model is applicable at different stages of PCVE’s impact on CUOSM.

Note: PCVE = previous cyberbullying victimization experiences, SMR = social media rumination, CUOSM = continuous use of social media.

**Table 2:** Statistical table of basic information from effective samples.

Statistical Items	Specific Content	Statistical Value	Percentage
Gender	Male	333	48.1%
	Female	359	51.9%
Age	18–26	185	26.7%
	27–40	337	48.7%
	41–55	143	20.7%
	Over 55	27	3.9%
Educational Background	High school and below	242	35.0%
	Undergraduate	425	61.4%
	Masters and doctorate	25	3.6%
Net age	Less than 3 years	3	0.4%
	3–5 years	55	7.9%
	5–10 years	214	30.9%
	Over 10 years	420	60.7%

**Table 3:** Results of the reliability and convergent validity analysis.

	Items	Factor Loading	KMO	AVE	CR	McDonald’s ω
1. PCVE	PCVE1	0.815	0.654	0.454	0.711	0.835
	PCVE2	0.718				
	PCVE3	0.801				
	PCVE4	0.833				
	PCVE5	0.840				
2. SMR	SMR1	0.856	0.818	0.591	0.852	0.900
	SMR2	0.860				
	SMR3	0.778				
	SMR4	0.843				
	SMR5	0.830				
3. Distress	Distress1	0.850	0.721	0.617	0.829	0.897
	Distress2	0.853				
	Distress3	0.886				
4. CUOSM	CUOSM1	0.796	0.750	0.435	0.705	0.819
	CUOSM2	0.756				
	CUOSM3	0.708				
	CUOSM4	0.633				

Note: PCVE = previous cyberbullying victimization experiences, SMR = social media rumination, CUOSM = continuous use of social media.

**Table 4:** Results of the discriminant validity analysis.

	1	2	3	4
1. PCVE	**0.669**			
2. SMR	0.370	**0.766**		
3. Distress	0.426	0.505	**0.789**	
4. CUOSM	−0.137	0.157	−0.088	**0.655**

Note: The square root of the AVE is on the diagonal and in bold, while the correlation coefficients of the variables analyzed are below it; PCVE = previous cyberbullying victimization experiences, SMR = social media rumination, CUOSM = continuous use of social media.

**Table 5:** Means, standard deviations, range, kurtosis, skewness, and correlations among variables.

Variables	M	SD	Range	Kurtosis	Skewness	1	2	3	4
1. PCVE	4.628	1.135	5.750	−0.036	−0.556	1			
2. SMR	3.912	1.385	5.670	−0.800	−0.117	0.370 ***	1		
3. Distress	4.872	1.280	5.750	0.476	−0.913	0.426 ***	0.505 ***	1	
4. CUOSM	2.439	0.831	6.000	0.887	0.740	−0.137 ***	0.157 ***	−0.880 *	1

Note: *** *p* < 0.001, * *p* < 0.05; PCVE = previous cyberbullying victimization experiences, SMR = social media rumination, CUOSM = continuous use of social media.

**Table 6:** Hierarchical quadratic regression analyses for PCE predicting CUOSM.

	Model 1		Model 2	
	B	*p*	B	*p*
Gender	−0.022	0.551	−0.006	0.876
Age	−0.109	0.004 **	−0.074	0.045 *
Educational background	−0.039	0.300	−0.030	0.413
Net age	−0.046	0.219	−0.040	0.269
PCVE	−0.124	0.001 **	−0.250	0.000 ***
PCVE^2^			−0.310	0.000 ***
R^2^	0.036		0.115	
Δ R^2^	0.029		0.108	

Note: *** *p* < 0.001, ** *p* < 0.01, * *p* < 0.05. PCVE = previous cyberbullying victimization experiences.

**Table 7:** Multiple regression results of the low PCE group.

Independent Variable	β	SE	*t*	*p*	R^2^	F
Dependent Variable: Social Media Rumination (SMR)						
PCVE (low)	0.741	0.137	5.400	0.000 ***	0.164	29.165 ***
Dependent Variable: Distress						
PCVE (low)	0.188	0.162	1.159	0.248	0.315	34.028 ***
SMR	0.621	0.089	7.001	0.000 ***		
Continuous Use of Social Media (CUOSM)						
PCEV (low)	0.348	0.123	2.835	0.005 **	0.174	10.346 ***
SMR	0.280	0.078	3.636	0.000 ***		
Distress	−0.132	0.062	−2.132	0.035 *		

Note: *** *p* < 0.001, ** *p* < 0.01, * *p* < 0.05. PCVE = previous cyberbullying victimization experiences, SMR = social media rumination, CUOSM = continuous use of social media.

**Table 8:** Results of mediating effect test (low PCVE group).

	Route	Effect	95% Boot CI	
			LLCI	ULCI
Indirect effect	PCVE—SMR—CUOSM	0.207	0.087	0.368
	PCVE—Distress—CUOSM	−0.025	−0.094	0.029
	PCVE—SMR—Distress—CUOSM	−0.061	−0.154	0.005
Total indirect effect		0.122	0.005	0.259

Note: PCVE = previous cyberbullying victimization experiences, SMR = social media rumination, CUOSM = continuous use of social media.

**Table 9:** Multiple regression results of the high PCVE group.

Independent Variable	β	SE	*t*	*p*	R^2^	F
Dependent Variable: Social Media Rumination (SMR)						
PCVE (high)	0.330	0.079	4.183	0.000 ***	0.031	17.493 ***
Dependent Variable: Distress						
PCVE (high)	0.313	0.061	5.180	0.000 ***	0.216	74.099 ***
SMR	0.323	0.033	9.923	0.000 ***		
Continuous Use of Social Media (CUOSM)						
PCVE (high)	−0.374	0.044	−8.496	0.000 ***	0.177	38.486 ***
SMR	0.150	0.025	5.954	0.000 ***		
Distress	−0.115	0.031	−3.739	0.000 ***		

Note: *** *p* < 0.001; PCVE = previous cyberbullying victimization experiences, SMR = social media rumination, CUOSM = continuous use of social media.

**Table 10:** Results of mediating effect test (high PCVE group).

	Route	Effect	95% Boot CI	
			LLCI	ULCI
Indirect effect	PCVE—SMR—CUOSM	0.049	0.022	0.084
	PCVE—Distress—CUOSM	−0.036	−0.065	−0.013
	PCVE—SMR—Distress—CUOSM	−0.012	−0.024	−0.004
Total indirect effect		0.001	−0.035	0.038

Note: PCVE = previous cyberbullying victimization experiences, SMR = social media rumination, CUOSM = continuous use of social media.

**Table 11:** Results of hypotheses and the research question.

Hypotheses	Contents	Results
H1	There is an inverted U-shaped curvilinear correlation between PCVE and CUOSM	Valid
H2a	SMR mediates (+) the relationship between PCVE and CUOSM	Valid
H2b	SMR mediates (+) the relationship between PCVE and distress	Partially valid
H3a	Distress mediates (−) the relationship between PCVE and CUOSM	Partially valid
H3b	Distress mediates (−) the relationship between SMR and CUOSM	Partially valid
H4	SMR and distress play chain-mediating roles in the influence of PCVE on CUOSM	Partially valid
RQ	Whether the model is applicable at different stages of PCVE’s impact on CUOSM.	Applicable for the right curve, Partially applicable for the left curve.

Note: PCVE = previous cyberbullying victimization experiences, SMR = social media rumination, CUOSM = continuous use of social media.

## Data Availability

Not Applicable.
